# Diagnostic values of noradrenaline administered dose, procalcitonin (PCT) and blood lactic acid for septic shock

**DOI:** 10.5937/jomb0-53853

**Published:** 2025-03-21

**Authors:** Lili Ding, Mengru Liu, Haijun Sun

**Affiliations:** 1 Suqian First Hospital, Department of Critical Care Medicine, Suqian, China

**Keywords:** sepsis, septic shock, noradrenaline, vasopressors, mortality, prognosis, intensive care unit, APACHE II Score, SOFA Score, PCT levels, epsa, septički šok, noradrenalin, vazopresori, smrtnost, prognoza, odeljenje intenzivne nege, APACHE II skor, SOFA skor, nivoi PCT

## Abstract

**Background:**

Sepsis/septic shock is a life-threatening condition that requires prompt and effective treatment. Noradrenaline is a widely used vasopressor to manage septic shock, but its optimal dosage remains unclear. This study aimed to investigate the effects of noradrenaline doses on the prognosis of patients with sepsis/septic shock and identify the influencing factors for patient survival.

**Methods:**

A retrospective study was conducted on 126 patients with sepsis/septic shock who received noradrenaline treatment in the intensive care unit (ICU). Patients were divided into survival (n=91) and death (n=35) groups. Clinical data, laboratory results, and noradrenaline doses were collected and compared between the two groups.

## Abstract (sr)

**Uvod:**

Sepsa/septički šok je stanje opasno po 'život koje zahteva brzo i efikasno lečenje. Noradrenalin je široko korišćen vazopresor za upravljanje septičkim šokom, ali njegova optimalna doza ostaje nejasna. Ova studija je imala za cilj da istrazi efekte doza noradrenalina na prognozu pacijenata sa sepsom/septičkim šokom i identifikuje faktore koji utiču na preživljavanje pacijenata.

**Metode:**

Sprovedena je retrospektivna studija na 169 pacijenata sa sepsom/septičkim šokom koji su primali terapiju noradrenalinom na odeljenju intenzivne nege (ICU). Pacijenti su podeljeni u dve grupe: preživeli (n=91) i preminuli (n=35). Prikupljeni su i upoređeni klinički podaci, laboratorijski rezultati i doze noradrenalina između ove dve grupe.

**Rezultati:**

Rezultati su pokazali da su visoke doze nor adrenalina bile povezane sa povećanom smrtnošću. Maksimalna i kumulativna doza noradrenalina su bile značajno više u grupi preminulih u poređenju sa grupom preživelih (P<0,05). Multivarijantna logistika analiza identifikovala je visoke nivoe PCT-a, visoke APACHE II i SOFA skorove, kao i visoke doze noradrenalina kao nezavisne faktore rizika za smrt. ROC analiza je otkrila je da je optimalna granična vrednost za maksimalnu dozu noradrenalina 0,792 mg/(kg·min), sa osetljivo{}u od 79,90% i specifičnošću od 69,28%. Kaplan-Majerova analiza preživljavanja pokazala je da su pacijenti u grupi sa nižim dozama imali višu stopu preživljavanja i duže srednje vreme preživljavanja u poređenja sa onima u grupi sa visokim dozama (P<0,05).

**Zaključak:**

Ova studija ukazuje da su visoke doze noradrenalina povezane sa lošom prognozom kod pacijenata sa sepsom/septičkim šokom. Optimalna doza noradrenalina treba pažljivo da se titrira kako bi se smanjio rizik od smrtnog ishoda. Potrebne su dalje studije kako bi se potvrdili ovi nalazi i utvrdila optimalna doza noradrenalina u upravljanju septičkim šokom.

## Introduction

Sepsis is a systemic inflammatory response syndrome caused by infectious pathogenic microorganisms. Patients with severe sepsis suffer from irreversible hypotension due to insufficient organ perfusion, which is also known as clinical septic shock. It is a major challenging symptom in treating critical diseases [1]. According to related statistical results, more than 19 million people suffer from sepsis, and about 6 million of the patients die each year around the world. The mortality is as high as 31%. Among all dead patients, about 40% die of septic shock. Besides, about 23% of 13 million survivors still suffer from cognitive dysfunction [2] [3] [4]. At present, clinical experts pay close attention to the influencing factors for the prognosis among patients with sepsis/septic shock. In addition, some relevant studies demonstrated that patients’ age, basic diseases, mannose-binding lectin 2 (MBL2), and high acute physiology and chronic health evaluation II (APACHE II) affected the prognosis among patients with septic shock [5] [6]. Septic shock originates from sepsis. Clinical observation showed that sepsis still aggravated even during bundle therapy guided by surviving sepsis campaign (SSC) guideline [7], and hypotension among patients with septic shock was caused by the reduced reaction of blood volume and body to noradrenaline [8]. Besides, some studies revealed that low-dose noradrenaline could improve left ventricular afterload in the early stage of septic shock [9]. Nevertheless, noradrenaline became the major clinical treatment method if hypotension still occurred after the rehabilitation of blood volume among patients with sepsis (high-dose therapy (0.5~2.0 mg/(kg·min))was commonly adopted) [10]. However, recent studies [11] showed that 1 mg/(kg·min) was high-dose noradrenaline. The relationship between noradrenaline dosing and septic shock mortality has been a topic of interest in recent studies. According to a prospective, observational cohort study [12], the use of norepinephrine as part of hemodynamic management may influence outcomes favourably in septic shock patients, contradicting the notion that norepinephrine potentiates end-organ hypoperfusion, thereby contributing to increased mortality. However, another study [13] found no statistically significant difference in 28-day mortality between patients with septic shock treated with high-dose norepinephrine compared with those treated with low-dose norepinephrine.A study on prehospital norepinephrine administration [14] reported that prehospital norepinephrine infusion to reach a mean blood pressure (MAP) >65 mmHg at the end of the prehospital stage is associated with a decrease in 30-day mortality in patients with septic shock. In contrast, a study on the impact of norepinephrine dose reporting heterogeneity on mortality prediction in septic shock patients [15] found that heterogeneous reporting of NE doses significantly affects mortality prediction in septic shock, and standardising NE dose reporting as base molecule could enhance risk stratification and improve processes of care. Nonetheless, no consensus has been reached on the clinical selection of the dose of noradrenaline. As the existing literature on sepsis/septic shock management highlights the uncertainty surrounding the optimal dosage of noradrenaline, with conflicting findings on its impact on mortality, and given the lack of consensus on the clinical selection of noradrenaline doses, we aimed to investigate the effects of different noradrenaline doses on the prognosis of patients with sepsis/septic shock along with the PCT levels and identify the influencing factors for patient survival. This study is novel in that it comprehensively analyses the relationship between noradrenaline dosing and septic shock mortality and sheds light on the optimal dosage of noradrenaline for improving patient outcomes.

## Materials and methods

### Research objects

This was a retrospective study on 169 patients clinically diagnosed with sepsis/septic shock at the critical care medicine department in our hospital between March 2020 and August 2023.

The patients enrolled in this work had to satisfy all the following conditions:

- A. All patients were diagnosed based on the Guidelines for Emergency Treatment of Sepsis/Septic Shock in China [16].
- B. All patients were aged over 18.
- C. Patients stayed in the intensive care unit (ICU) for 72 hours or longer.
- D. All patients with persisten thypotension under went the treatment of noradrenaline.

The patients with any of the following conditions had to be excluded:

- A. Patients suffered from cardiogenic or hemorrhagic shock.
- B. Patients were pregnant or at lactation period.
- C. Patients suffered from severe cardiovascular and cerebrovascular diseases.
- D. Patients who were treated with noradrenaline for less than 24 hours.
- E. Patients who lost some general clinical data and treatment data.

The implementation of this research had been approved by the Medical Ethics Committee (Ethics No. 20230028).

### Research methods

A retrospective research method was adopted for the comparison and analysis of the clinical data on the patients who were transferred to the general ward or discharged after the disease became stable (survival group, n=91 cases), those who were voluntarily discharged from ICU, and dead patients (death group, n=35 cases). Their clinical data were collected and compared to explore the effects of the factors influencing the prognosis among patients with septic shock and different doses of noradrenaline on patient prognosis.

The clinical data on the 1^st^ day of hospitalisation were set as initial data.

The general clinical data on patients were collected, including gender, age, sequelae, whether shock occurred, infected sites (lung infection, abdominal infection, urinary tract infection, bloodstream infection, central nervous system infection, and infection at other sites), bacterial species (Gram-positive bacterium (G ^+^ bacterium), Gram-negative bacterium (G ^-^ bacterium), fungus, or mixed bacterium), and whether primary diseases occurred (coronary heart disease, chronic obstructive pulmonary disease (COPD), diabetes, hypertension, cancer, and hepatic renal insufficiency).

Blood samples were collected from patients. White blood cell (WBC) count, procalcitonin (PCT), blood lactic acid, serum creatinine (Scr), platelet count, and oxygen saturation were measured at four time points: on admission to ICU (T0), and 12–24 hours (T1), 25–36 hours (T2), and 37–48 hours (T3) after initiation of treatment. Blood samples were collected in EDTA tubes and sent to the laboratory for analysis within 30 minutes of collection. An automated haematology analyser (Sysmex XN-9000) measmeasured WBC and platelet counts. In contrast, PCT, lactic acid, and Scr were measured using a chemiluminescence immunoassay (Roche Cobas e602) and an automated biochemistry analyser (Roche Cobas c702), respectively. Oxygen saturation was measured using a pulse oximeter (Masimo Radical-7).

APACHE II [17] scores and initial systemic infection sepsis-related organ failure assessment (SOFA) [18] scores (respiration system, blood coagulation, liver, cardiovascular system, central nervous system, and kidney) on admission to ICU were collected.

The daily dose, maximum dose, and cumulative dose of noradrenaline were collected.

### Statistical methods

SPSS 22.0 was utilised for data statistical analysis. Measurement data conforming to normal distribution were expressed as mean±standard deviation, while measurement data that did not conform to normal distribution were denoted by median (quartile) [M (*P*_*25*_, *P*_*75*_)]. Enumeration data were recorded as n (%), and measurement data conforming to the normal distribution in different groups were compared using an independent sample t-test, while nonnormal enumeration data were compared using the Mann- Whitney test. Besides, enumeration data were compared using the Chi-square test. Logistic regression analysis was carried out to investigate factors influencing patient survival. The receiver operating characteristic curve (ROC) was drawn, and the area under the curve (AUC) was used to quantitatively analyse the prognostic assessment values of different doses of noradrenaline for patients with septic shock. ROC analysis determined the optimal nodal value, sensitivity, and specificity. In addition, Kaplan-Meier survival analysis was adopted to compare the survival rates of patients treated with different doses of noradrenaline. =0.05 served as the test level, and *P*<0.05 indicated that the difference suggested statistical significance.

## Results

### Statistics on general clinical data

In this research, basic clinical data on 91 patients in the survival group and 35 patients in the death group were summarised and compared, including gender, age, whether shock occurred, infected sites, bacterial species, and basic diseases (Table 1). The average age among patients in the survival and death groups amounted to 67.23±11.44 and 65.11±13.02, respectively (*P*>0.05). No apparent statistical differences were visualised in gender ratio and the proportions of patients with shock, lung infection, abdominal infection, urinary tract infection, bloodstream infection, central nervous system infection, infection at other sites, G^+^ bacterium, G^-^ bacterium, fungal infection, mixed bacterial infection, coronary heart disease, diabetes, and renal insufficiency between patients in survival and death groups (*P*>0.05). The proportions of patients with hypertension, COPD, cancer, and hepatic insufficiency were notably lower in the survival group, exhibiting great differences from those in the death group (*P*<0.05).

**Table 1:** Summary basic clinical data on patients. Notes: * suggested that differences were detected in patients in survival and death groups (*P*<0.05).

Items		Survival group<br>(n=91 cases)		Death group<br>(n=35 cases)		P
		Number<br>(cases)	Proportion<br>(%)	Number<br>(cases)	Proportion<br>(%)	
Males		55	60.44	21	60	0.123
Females		36	39.56	14	40	0.232
Shock		57	62.64	25	71.43	0.111
Infected<br>sites	Lung infection	20	21.98	14	40	0.092
	Lung infection	27	29.67	21	60.00	0.082
	Urinary tract infection	16	17.58	9	25.71	0.091
	Bloodstream infection	20	21.98	10	28.57	0.067
	Infection at other sites	8	8.79	13	31.14	0.060
Infectious<br>bacterial<br>species	G+ bacterial infection	16	17.58	2	5.71	0.113
	G- bacterial infection	46	50.55	1	2.86	0.088
	Fungal infection	6	6.59	8	22.86	0.089
	Mixed bacterial infection	23	25.27	17	48.57	0.063
Basic<br>diseases	Coronary heart disease	49	53.85	2	5.71	0.134
	Hypertension	61	67.03	8	22.86	0.021*
	Diabetes	40	43.96	18	51.43	0.221
	COPD	46	50.55	32	91.43	0.033*
	Cancer	16	17.58	17	48.57	0.023*
	Hepatic	27	29.67	22	62.86	0.003*
	Renal insufficiency	11	12.09	11	31.43	0.051

### Statistics on laboratory examination results

**Figure 1:**
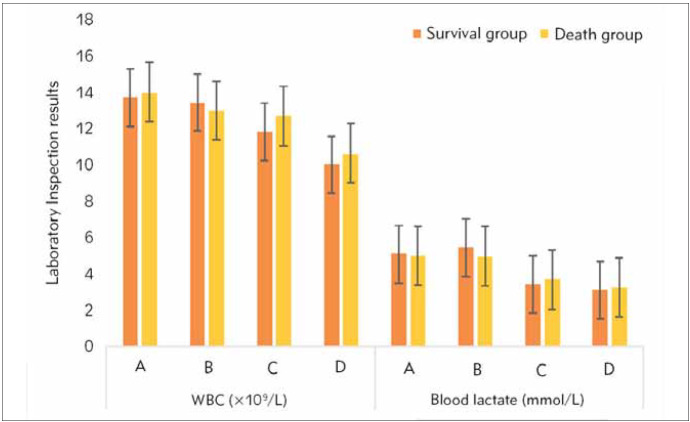
WBC count and blood lactic acid. A. On admission to ICU. B. 12h to 24h after treatment. C. 25h to 36h after treatment. D. 37h to 48h after treatment.

**Figure 2:**
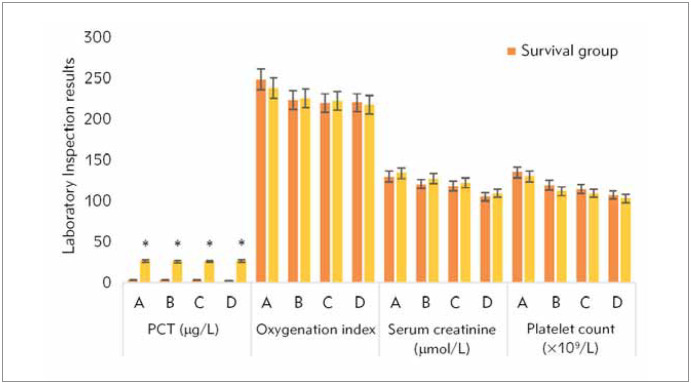
Cr, Scr, platelet count, and oxygen saturation. A. On admission to ICU. B. 12h to 24h after treatment. C. 25h to 36h after treatment. D. 37h to 48h after treatment. * suggested that the differences were significant (P<0.05).

WBC count, C reactive (Cr), blood lactic acid, Scr, platelet count, and oxygen saturation among patients in the survival group and death group on the admission to ICU and 12h to 24h, 25h to 36h, and 37h to 48h after treatment were summarised (Figure 1 and Figure 2). It was demonstrated that no remarkable statistical differences were detected in the above indicators (except for Cr) among patients in the two groups in the above periods (*P*>0.05). The PCT levels in the survival group patients within the first 12 to 48 hours of ICU admission and treatment were sharply lower than those in the death group, with *P*<0.05.

### Statistics on other data

### A. Statistics on APACHE II and SOFA scores

**Figure 3:**
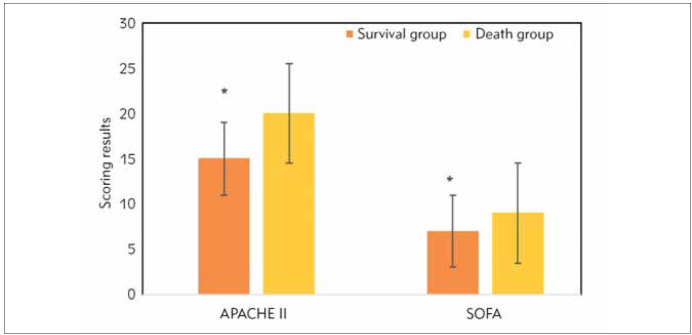
Comparison of APACHE II and SOFA scores for patients between the two groups. * suggested that the differences were significant (P<0.05).

APACHE II and SOFA scores for patients in the survival and death groups on admission to the ICU were summarised and compared (Figure 3). APACHE II and SOFA scores of the survival group and death group amounted to 15.00±4.47 vs 20.00±5.63 and 7.00±2.91 vs 9.00±2.98, respectively. The above two scores for survival group patients were lower (*P*<0.05).

### B. Statistics on the dose of noradrenaline

**Figure 4:**
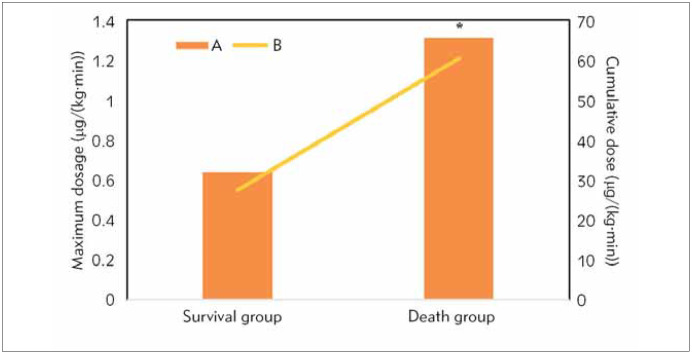
Dose of noradrenaline for patients in various groups A. Maximum dose. B. Cumulative dose. * suggested that the differences were significant (P<0.05).

The daily dose of noradrenaline for patients in the survival and death groups was collected to determine the maximum dose and cumulative dose, as demonstrated in Figure 4. It was demonstrated that the maximum dose and cumulative dose for patients in the survival group and death group amounted to 0.64 μg/(kg·min)(0.40~1.20) vs 1.31(0.70~2.00) and 27.33 μg/(kg·min)(13.00~79.00) vs 60.42 μg/(kg·min)(47.00~153.00), respectively. The above doses for patients in the survival group were apparently lower and demonstrated a sharp difference from those in the death group (*P*<0.05).

### Univariate and multivariate logistic analysis (ULA) and (MLA)

ULA and MLA were performed for the prognostic factors for patients with sepsis/septic shock (Table 2). According to ULA, basic diseases (hypertension, COPD, cancer, and liver dysfunction), PCT, APACHE II and SOFA scores, and maximum dose and cumulative dose of noradrenaline affected the treatment and prognosis for patients (*P*<0.05).

**Table 2:** ULA and MLA of the prognosis for patients with sepsis/septic shock. Note: OR referred to odds ratio and * suggested that the outcomes demonstrated statistical differences (P<0.05).

Indicators	ULA			MLA		
	*OR*	*95%CI*	*P*	*OR*	*95%CI*	*P*
Hypertension	2.021	1.671~2.871	0.021*	1.891	1.222~2.276	0.089
COPD	1.871	1.091~2.312	0.033*	1.923	1.438~2.274	0.078
Cancer	2.091	1.212~2.561	0.023*	1.762	1.207~2.038	0.213
Liver dysfunction	1.213	0.871~1.781	0.003*	1.671	1.034~1.982	0.113
PCT	1.991	1.099~2.562	0.002*	1.341	0.982~1.762	0.033*
APACHE	1.391	0.794~1.873	0.001*	2.471	1.852~2.893	0.001*
SOFA	1.651	0.999~2.206	0.003*	2.651	2.091~2.998	0.021*
Maximum dose of noradrenaline	1.471	0.781~1.993	0.034*	2.191	1.761~2.769	0.002*
Cumulative dose of noradrenaline	1.234	0.761~1.832	0.009*	2.130	1.726~2.541	0.007*

Based on ULA results, the above indicators were set as variables for MLA. It was demonstrated that high PCT levels, high APACHE II and SOFA scores, and high doses of noradrenaline were the independent risk factors for death among patients with sepsis and septic shock (*P*<0.05).

### ROC assessment

**Figure 5:**
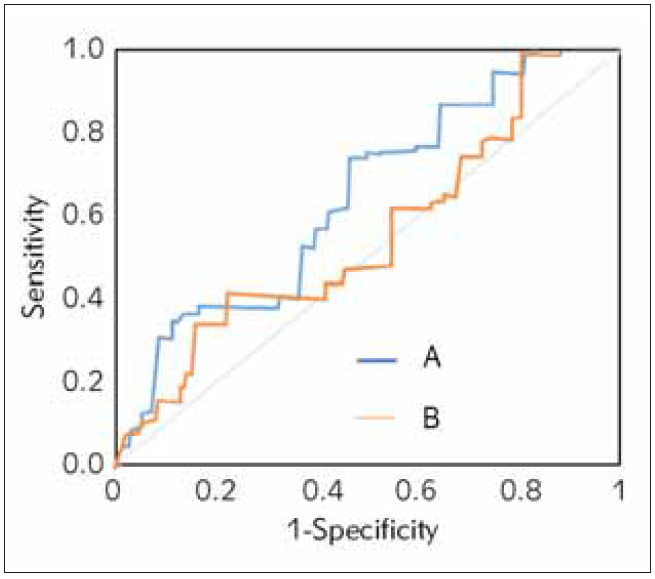
ROCs of maximum dose (B) and cumulative dose (A) of noradrenaline.

ROCs were drawn for maximum dose and cumulative dose of noradrenaline (Figure 5). It was suggested that the prediction effect was the best when the AUC of ROC of the maximum dose amounted to 0.771. Besides, the corresponding dose, sensitivity, and specificity were 0.792 μg/(kg·min), 79.90%, and 69.28%, respectively. When the AUC of ROC of cumulative dose was 0.809, the prediction effect was the best. When the corresponding total dose amounted to 47 mg, the predictive sensitivity and specificity for death among patients with septic shock were 88.59% and 70.27%, respectively.

### Comparison of the survival rate among patients with different doses

**Figure 6:**
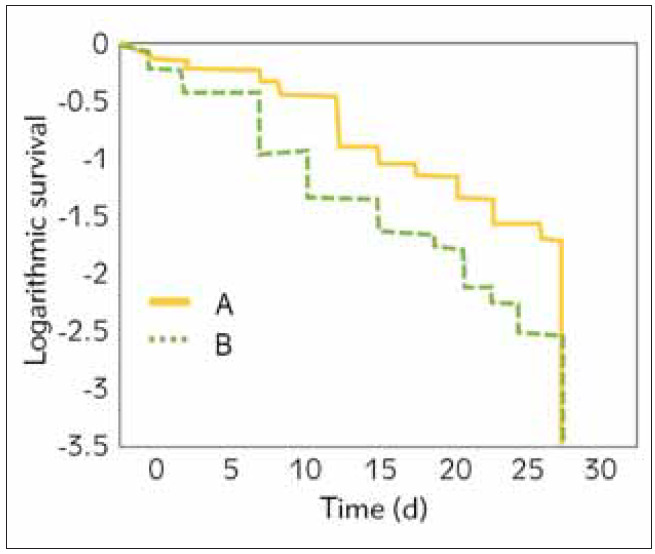
Comparison of Kaplan-Meier survival curves of different doses of noradrenaline A. Low-dose group. B. High-dose group

The maximum dose of noradrenaline obtained in the above research (0.792 μg/(kg·min)) was set as the node. The included patients were divided into a high-dose group ( 0.792 μg/(kg·min, n=47 cases) and a low-dose group (<0.792 μg/(kg·min, n=79 cases). Kaplan-Meier survival analysis was carried out to compare the survival rates among patients treated with different doses of noradrenaline (Figure 6). It was found that the median survival time (MST) was 9.56(5.23~28.00) d and 20.67(7.62~28.00) d among patients in the high-dose group and low-dose group, respectively. MST among patients in the latter group was higher than in the former group (*P*<0.05). In addition, the survival rate among patients in the low-dose group was higher than that among patients in the high-dose group at any time.

## Discussion

Our study found that high PCT levels were associated with increased mortality in sepsis patients, which is consistent with the findings of several other studies [19] [20] [21]. For example, a systematic review and meta-analysis published in the Lancet Infectious Diseases found that PCT was a significant predictor of mortality in sepsis patients [21]. Another study published in Critical Care found that PCT levels were higher in non-survivors than in sepsis survivors [20]. However, our study also found that PCT was not the only predictor of mortality and that other factors, such as APACHE II and SOFA scores and the dose of noradrenaline, also played a role. This is consistent with the findings of other studies, which found that PCT is just one of several biomarkers that can predict mortality in sepsis patients 4. In terms of the diagnostic accuracy of PCT, our study found that it had a high sensitivity and specificity for predicting mortality, which is consistent with the findings of other studies [19] [20]. However, the study also found that PCT’s predictive value was improved when combined with other biomarkers, such as C-reactive protein and interleukin-6 [22].

Our study investigated the effect of noradrenaline dose on mortality in patients with sepsis and septic shock. In contrast, a randomised controlled trial [23] compared the effects of dopamine and epinephrine as first-line vasoactive drugs in pediatric septic shock, finding no significant difference in 28-day mortality. Another study [24] compared epinephrine alone with norepinephrine plus dobutamine in patients with septic shock, also finding no difference in efficacy and safety. A network meta-analysis [25] of randomised trials found that vasoactive drugs, including epinephrine and norepinephrine, had no significant effect on mortality in patients with severe sepsis and septic shock. However, a retrospective cohort study [26] found that epinephrine administration in venoarterial extracorporeal membrane oxygenation patients was associated with increased mortality.

Our study found that high PCT levels, high APACHE II and SOFA scores, and high doses of noradrenaline were independent risk factors for death among patients with sepsis and septic shock. In contrast, Freund et al. [27] found that the qSOFA score had better but only moderate accuracy in predicting mortality in elderly and very elderly sepsis patients. The study by Jiang et al. [28] found that the qSOFA score was not superior to the SIRS criteria in predicting mortality in infected patients in the emergency department. The study by Sanguanwit et al. [29] developed a clinical score for predicting 28-day mortality in geriatric sepsis patients, which included factors such as age, comorbidities, and laboratory results.

In contrast to our study, a study by Macdonald et al. [30] found that the PIRO score was a better predictor of mortality in emergency department patients with severe sepsis and septic shock. Another study by Songsangjinda et al. [31] compared the performance of different severity score models in predicting in-hospital mortality among sepsis patients in the ICU and found that the APACHE II score was a good predictor of mortality. Our study also found that the maximum dose and cumulative dose of noradrenaline were associated with mortality, which is consistent with the findings of a study by Aggrawal et al. [32] that found that the dose of noradrenaline was a predictor of mortality in patients with septic shock. However, our study found that the PCT level was a stronger predictor of mortality than the APACHE II score, which is in contrast to the findings of a study by Wang et al. [33] that found that the APACHE II score was a better predictor of mortality than the PCT level.

With the continuous social ageing, the constant rise of the prevalence of cancer, and the constant emergence of various invasive surgical methods, the prevalence of sepsis also increases year by year, and the fatality rate remains high [34]. To understand the influencing factors for the prognosis for patients with sepsis/septic shock, 168 patients with sepsis/septic shock were selected as the subjects and enrolled into the survival group and death group to analyse the influencing factors for prognosis. The research findings demonstrated that there were remarkable differences in the occurrence of basic diseases (hypertension, COPD, cancer, and liver dysfunction), PCT level, the scores for APACHE II and SOFA, and the maximum and cumulative doses of noradrenaline between patients in survival group and death group (*P*<0.05). ULA also revealed that the above indicators affected the prognosis among patients with sepsis/septic shock. It was suggested that hypoimmunity occurred among patients with liver dysfunction, hypertension, and COPD [35] [36] and radiochemotherapy, immuno suppression, and other therapies for tumour diseases also deteriorate patients’ overall nutritional status [37]. Consequently, patients were more likely to suffer from severe cachexia. However, MLA demonstrated that hypertension, COPD, cancer, and liver dysfunction occur as independent risk factors for death among patients (*P*>0.05). Petrovic et al. [38] showed that the prognosis for patients with critical sepsis was closely associated with calcitonin level while unrelated to non-specific Cr. In contrast, Kanashvili et al. [39] pointed out that Cr could play a vital role in the early assessment and management of multiple trauma-induced sepsis and septic shock as a non-specific indicator for the severity of pathological progression, which was consistent with the research finding that high PCT was an independent risk factor for death among patients. It was found that both APACHE II and SOFA scores could be utilised for the prognostic assessment of patients with septic shock in ICU, supported by relevant research [40] [41]. The research findings showed high APACHE II and SOFA scores were associated with patient prognosis. Colussi et al. [42] and Innocenti et al. [43] also suggested that APACHE II and SOFA scores could predict the prognosis effectively.

In addition, it was demonstrated that the patient’s prognosis was related to the dose of noradrenaline. According to the relevant study, both maximum and cumulative doses of noradrenaline for patients in the death group (1.31 and 60.42 μg/(kg·min)) were superior to those for patients in the survival group (0.64 and 27.33 μg/(kg·min)) (*P*<0.05), which might be caused by the reduced reaction of patients to noradrenaline [40]. Besides, some research demonstrated that the dose of noradrenaline was associated with the prognosis for patients with septic shock [44] [45]. What is more, ROC analysis showed that the maximum dose, sensitivity, and specificity of noradrenaline amounted to 0.792 μg/(kg·min), 79.90%, and 69.28%, respectively, which revealed that its maximum dose could be regarded as the prospective indicator for prognostic assessment [46]. Besides, the maximum dose was set as a node, and it was found that MST was shorter among patients with a dose higher than 0.792 μg/(kg·min) (9.56 d) than among those with a lower dose (20.67 d) while the mortality was higher. In other words, the continuous increase of the dose of noradrenaline per unit of time would lead to a rise in mortality when blood pressure could not be stabilised by a low dose and normal dose of noradrenaline.

## Conclusion

In this research, a retrospective survey and analysis were implemented to influence the prognosis among patients with sepsis and septic shock within 28 days after admission to the ICU. Besides, the application values of the dose of noradrenaline in prognostic assessment were investigated. The research findings demonstrated that high PCT, high APACHE II and SOFA scores, and high doses of noradrenaline affected the prognosis among patients with sepsis/septic shock 37–48 h after the treatment. The dose of noradrenaline reflected the prognosis. The above research findings provided some basis for clinical physicians.

Nonetheless, this research was retrospective, which might be mixed with some unidentifiable and uncontrollable influencing factors. In addition, the research sample size was small, the research scope was not very extensive, and the research findings might not be representative enough. Future research should be more real-time, and the sample size and scope should be enlarged to provide more reliable results for clinical treatment.

## Dodatak

### List of abbreviations

APACHE II, Acute Physiology and Chronic Health Evaluation II;<br>COPD, Chronic Obstructive Pulmonary Disease;<br>Cr, C-reactive protein;<br>ICU, Intensive Care Unit;<br>MAP, Mean Arterial Pressure;<br>MBL2, Mannose-Binding Lectin 2;<br>MST, Median Survival Time;<br>NE, Norepinephrine;<br>OR, Odds Ratio;<br>PCT, Procalcitonin;<br>ROC, Receiver Operating Characteristic;<br>Scr, Serum Creatinine;<br>SOFA, Sepsis-Related Organ Failure Assessment;<br>SSC, Surviving Sepsis Campaign;<br>WBC, White Blood Cell.

### Conflict of interest statement

All the authors declare that they have no conflict of interest in this work.
